# Genetic variation in cows' response to methane-mitigating feed additives

**DOI:** 10.3168/jdsc.2025-0885

**Published:** 2025-11-13

**Authors:** Bj⊘rg Heringstad, Karoline A. Bakke

**Affiliations:** 1 Department of Animal and Aquacultural Sciences, Faculty of Biosciences, Norwegian University of Life Sciences, 1432 Ås, Norway; 2 Geno Breeding and AI Association, 2317 Hamar, Norway

## Abstract

Feed additives such as 3-nitrooxypropanol (3-NOP) can reduce a dairy cow's enteric methane (CH_4_) emissions. Our study aimed to examine whether there is genetic variation in the cow's response in reduction of CH_4_ emission after receiving 3-NOP feed additives. Data were available from a project in which the CH_4_-mitigating effect of 3-NOP (Bovaer, DSM-Firmenich) was tested for Norwegian dairy cows. The one-year trial took place in a commercial dairy herd and included 79 Norwegian Red cows. The cows' CH_4_ emissions were measured by a GreenFeed unit. A total of 14,166 daily CH_4_ measures were analyzed, and the overall mean (SD) was 387 (96) g of CH_4_ per cow per day. Cows were divided into 2 groups, where 54 cows received between 1.2 and 1.5 g of 3-NOP per day and 25 cows were in a control group without any feed additives. The trait of interest, CH_4_-response, was defined as the cow's change in CH_4_ after introducing 3-NOP in the diet compared with the base level CH_4_ in periods without 3-NOP. The base level period consisted of 3 wk in September of 2023 and 4 wk in January and February of 2024. The trait daily CH_4_ (g/d) was analyzed with a linear animal repeatability model with fixed effects of 3-NOP group, parity, lactation week, and test day, and random animal genetic and permanent environment effects. Fixed effect solutions from this model were used to compute yield deviations for CH_4_ (YD_CH_4_), a corrected phenotype (i.e., daily CH_4_ corrected for effects of parity, lactation stage, and test day). For cows fed 3-NOP, we calculated their individual base level CH_4_ as the average YD_CH_4_ from the periods without 3-NOP. The trait CH_4_-response was then calculated as YD_CH_4_ minus base level CH_4_. The new CH_4_-response trait had a total of 7,293 daily records from 42 cows and was analyzed with a linear animal repeatability model with the fixed effect of test day and random additive genetic effect of cow. Although this is a small dataset and results should be interpreted with caution, the estimated heritability of 0.15, with an SE of 0.03, suggests that genetic variation in response in the reduction of CH_4_ emission after receiving 3-NOP feed additives exists for Norwegian Red dairy cows.

## Highlights

• The methane-mitigating effect of feed additives varies between cows.
• There is genetic variation in the cows' response after receiving 3-NOP feed additives.
• Breeding cows that respond more to methane-mitigating feed additives seems possible.

## Graphical Abstract

**Figure:**
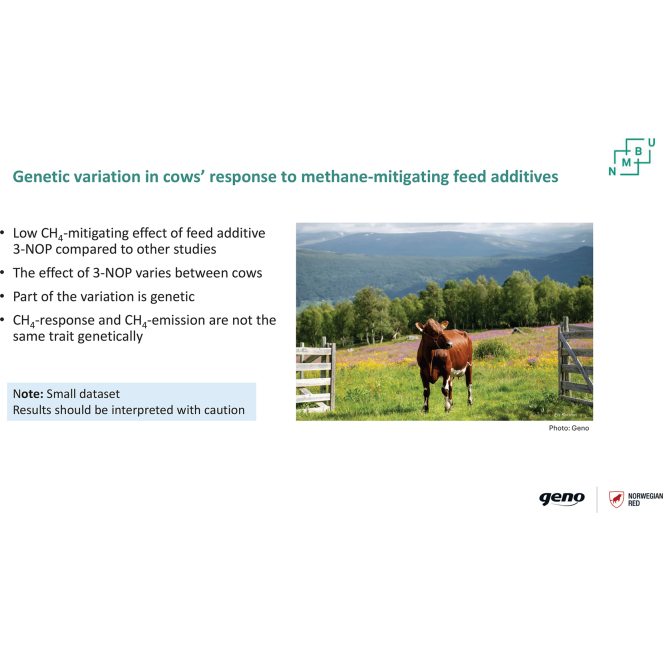
**Summary:** There is genetic variation between cows in how much feed additives can reduce methane (CH4) emissions. GreenFeed (C-Lock Inc.) measures of methane were available from a 1-year trial where the methane-mitigating effect of the feed additive 3-nitrooxypropanol (3-NOP; Bovaer, DSM-Firmenich) was tested for Norwegian Red dairy cows in a commercial herd. The overall effect of 3-NOP was an 8% reduction in methane emissions. The estimated heritability of 0.15 for response in reduced methane emissions suggests that it is possible to increase response to methane-mitigating feed additives by breeding.

Feed additives can reduce enteric methane (CH_4_) emissions from dairy cows. Several recent review papers (e.g., Hristov, 2024) and meta-analyses (e.g., Kebreab et al., 2023; Martins et al., 2024) have addressed methane mitigation practices in dairy production. They show that the CH_4_ mitigation effect of feed additives varies and is affected by diet, diet composition, nutritional value, feeding strategies, and production systems. The response to methane-mitigating feed additives might also vary among cows, and part of this variation may be genetic. We know that breeding for lower CH_4_ emissions is possible (e.g., Lassen and Difford, 2020), and a question of interest is whether it is also possible to breed cows that respond better to methane-mitigating feed additives. Our study aimed to examine whether there is genetic variation in the cow's response in the reduction of CH_4_ emission after receiving 3-nitrooxypropanol (**3-NOP**) feed additives.

Data were available from a project run by MetanHUB (www.metanhub.no) in which the CH_4_-mitigating effect of 3-NOP (Bovaer, DSM-Firmenich) was tested for Norwegian dairy cows during one year, spanning from June 2023 to June 2024. The trial took place in a commercial dairy herd and included a total of 79 Norwegian Red cows. Methane emissions were measured by a GreenFeed (**GF**) unit (C-Lock Inc.), where each visit provided an estimate of the animal's daily CH_4_ emission. The cow had to stay at least 2 min with correct head position for a GF visit to be accepted as good data. Records from 5 to 350 DIM and from parities 1 to 8 were included. Measures outside the range of 100 to 800 g of CH_4_ per day were excluded as outliers. These were the same editing criteria as previously used in analyses of Norwegian GF data, based on a combination of visual inspection of the distribution of phenotypic records and statistical detection of outliers. For each cow we computed daily CH_4_ as the average of her GF visits each day. In total 14,166 daily measures were included, and the overall mean (SD) was 387 (96) g of CH_4_ per cow per day. The trial had 2 groups, with 54 of the cows receiving between 1.2 and 1.5 g of 3-NOP per day, dispensed from a concentrate feeding station, and 25 cows in a control group without any feed additives. The group assignment aimed to balance the groups as far as possible with respect to parity and lactation stage. The proportion of cows in their first, second, and later lactations was 54%, 13%, and 33% in the 3-NOP group and 52%, 12%, and 36% in the control group, respectively. Both groups were kept together in the same freestall barn, with the same feed, feeding strategy, and management. Cows were fed grass silage ad libitum and received concentrates according to milk yield. Concentrates were fed both in the milking robot and in a feeding station. Both groups had access to the same feed bins for grass silage. The 2 groups had similar genetic levels. Comparing breeding values from Geno's (Hamar, Norway) routine genetic evaluation did not reveal any clear differences between the 2 groups. The difference between average breeding values in the 2 groups was in the range of 0 to 2 index points (standardized breeding values with mean 100 and SD 10) for most milk production, health, and fertility traits.

The overall mean (SD) of daily CH_4_ during the trial was 378 (96) g for the 3-NOP group and 409 (92) g for the control group. To study the response in the reduction of CH_4_ emission, we needed a measure of the base level CH_4_ individually for each cow. For this, we used information from periods without distribution of 3-NOP; 3 wk in September of 2023 and 4 wk in January and February of 2024. The GLM procedure in SAS 9.4 (SAS Institute, 2025) was used to check the significance of fixed effects and to compare LSM between groups. The DMU software (Madsen and Jensen, 2013) was used for genetic analyses. First, daily CH_4_ for all cows was analyzed with a linear animal repeatability model with fixed effects of 3-NOP group (2 groups), parity (1, 2, 3, >3), lactation week (1–50), and test day, and random animal genetic and permanent environment effects. The pedigree file was traced back 5 generations and included a total of 1,883 animals. The 2 groups had similar genetic levels for methane emission. The breeding values for CH_4_ (g/d) ranged from −24 to +49 in the control group and from −30 to +55 in the 3-NOP group, with averages of 3 and 0, respectively. Fixed effects solutions from this model were used to compute daily yield deviations for CH_4_ (**YD_CH_4_**). For each cow in the 3-NOP group, the individual base level CH_4_ was calculated as the average YD_CH_4_ from periods without 3-NOP, and the CH_4_-response for days with 3-NOP was the difference between the daily YD_CH_4_ and the cow's base level. Only cows with a base level calculated from records from at least 5 d were kept for further analyses. We had a total of 7,293 daily observations of CH_4_-response from 42 cows. The trait CH_4_-response was analyzed with a linear animal repeatability model with the fixed effect of test day and additive genetic effect of cow.

The distribution of CH_4_ measures for the 2 groups of cows is shown in Figure 1. The phenotypic level of daily CH_4_ was similar to previous reports for Norwegian Red dairy cows (Heringstad and Bakke, 2025). The LSM (SE) for daily CH_4_ was 377 (1.4) g/d for the 3-NOP group and 403 (1.8) g/d for the control group. The difference between groups indicates an overall mitigating effect of 3-NOP of around 30 g of CH_4_ per cow per day, or a reduction of ∼8% in CH_4_ emissions, which is low compared with other studies. In a recent review, Kebreab et al. (2023) reported that 3-NOP reduced enteric CH_4_ emissions in dairy cattle by more than 30% on average based on 14 experiments. Ma et al. (2024), who examined the effects of feed additives on CH_4_ emissions under Swiss management conditions, reported an average reduction in CH_4_ production (g/d) of 13% for cows supplemented with 3-NOP. Large differences in CH_4_ mitigating effects between studies can be explained by differences in diet composition, feeding strategies, and production systems. For example, van Gastelen et al. (2022) reported a more pronounced CH_4_ mitigation effect of 3-NOP in the corn silage–based diets than in the grass silage–based diets. Dairy production in Norway is based mainly on grass and grass silage, and this may explain the lower effect in the current study. Another reason for the lower effect could be that the time interval between feedings of 3-NOP was not regulated, as the cows visited the feeding station voluntarily.

**Figure 1:**
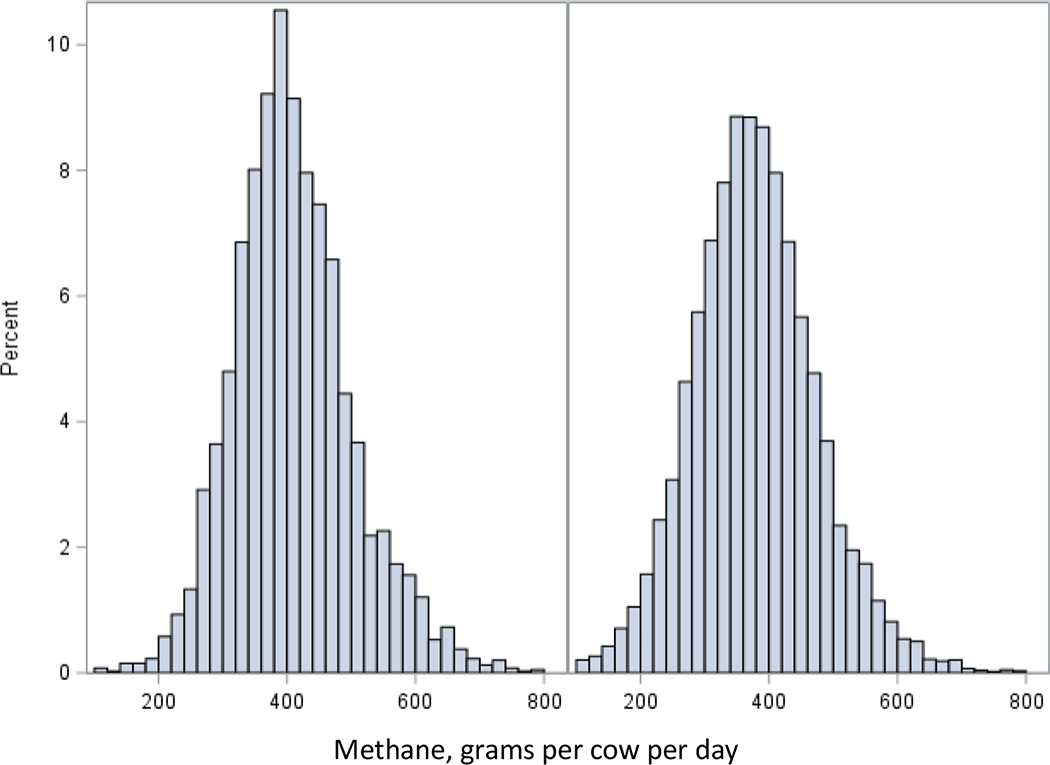
Distribution of daily methane measures (grams of CH_4_ per cow per day) in the control group (left) and the group that received 3-NOP feed additives (right).

The estimated variance components (SE) for CH_4_-response were 852 (182) and 4,933 (80) for additive genetic and residual effects, respectively, with a corresponding heritability (SE) of 0.15 (0.03). This is, as far as we know, the first study to estimate heritability of response to methane-mitigating feed additives.

The EBVs for CH_4_-response, given in grams CH_4_ per day, for cows with records ranged from −53 to +58 (Figure 2), indicating significant genetic differences between animals. Here, negative values are favorable, and lower values indicate a larger response to 3-NOP in the diet.

**Figure 2:**
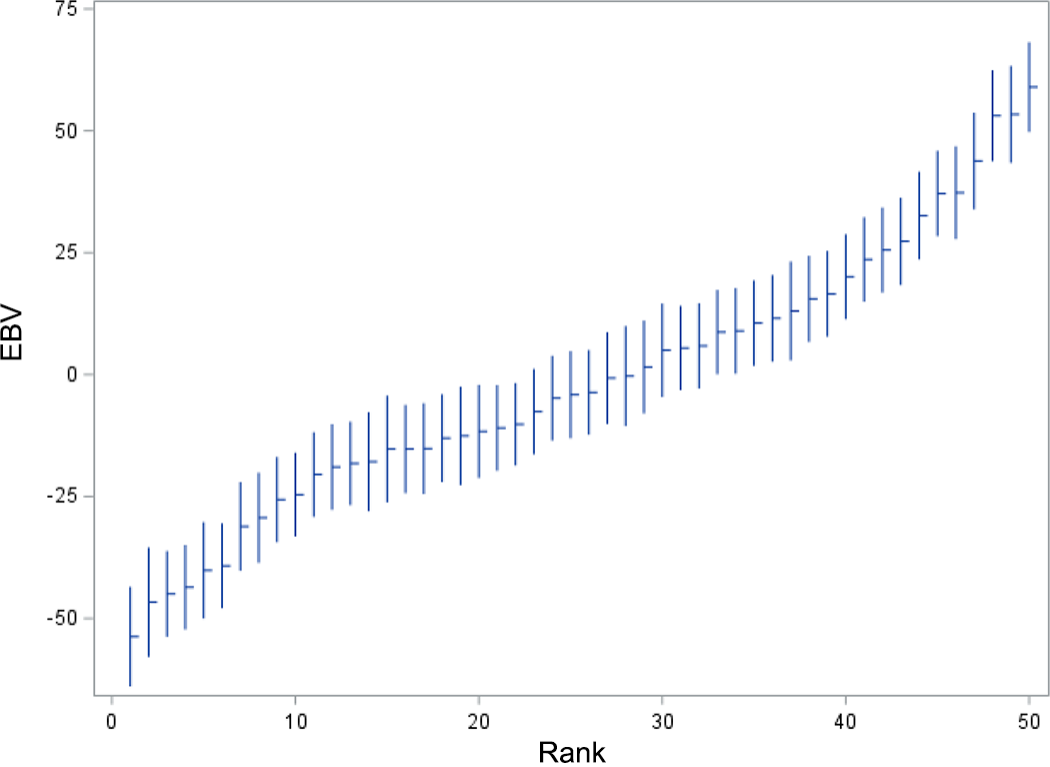
Estimated breeding values for CH_4_-response (whiskers indicate ±1 SE) for cows with records, sorted by increasing EBV for CH_4_-response.

Correlation between breeding values can indicate the strength and direction of the genetic correlation between traits. In our study, the correlation between EBV for CH_4_-response and EBV for CH_4_ emission (0.07) was not significantly different from 0, possibly indicating that the novel CH_4_-response trait is a genetically different trait.

Although this is a small dataset and results should be interpreted with caution, the estimated heritability of 0.15, with an SE of 0.03, suggests that genetic variation exists for response in the reduction of CH_4_ emission after receiving 3-NOP feed additives. Our results indicate that it would be possible to breed cows that respond more strongly to 3-NOP. However, it is probably not a good idea to breed for increased response to a specific feed additive. If our findings are supported by a larger dataset, it is important to be aware that genotype–environment interaction exists, and we may need to take this into account in our genetic evaluation models.
